# California’s Approach to the 2022 National Strategy to Support Family Caregivers

**DOI:** 10.1093/geroni/igaf122.996

**Published:** 2025-12-31

**Authors:** Rachael Fulp-Cooke, Janice Bell, Quynh Vo, Heather Young

**Affiliations:** Betty Irene Moore School Of Nursing at UC Davis, Sacramento, California, United States; Betty Irene Moore School Of Nursing at UC Davis, Sacramento, California, United States; University of California, Davis, Davis, California, United States; Betty Irene Moore School Of Nursing at UC Davis, Sacramento, California, United States

## Abstract

Family caregivers provide essential support for older adults and people with disabilities across California and the United States. The 2022 National Strategy to Support Family Caregivers (“National Strategy”) outlines five goals, 27 outcomes, and hundreds of actions that federal, state, and local governments, businesses, and communities can implement to strengthen caregiver support. To identify strengths and opportunities for expanding caregiving support in California in line with national priorities, we identified publicly funded caregiving initiatives in the state and assessed their alignment with the National Strategy. Caregiving initiatives were identified using the Data Dashboard for Aging, the Master Plan for Aging Implementation Tracker, the AARP Long-Term Services and Support Scorecard 2023, and additional internet research. Each initiative was mapped to one or more National Strategy outcomes, with results summarized in visual matrices.  Findings indicate that California demonstrates strong alignment with the National Strategy offering a diverse range of caregiving support initiatives. Key strengths include 1) a robust policy framework, 2) a statewide caregiver resource center network, 3) a centralized caregiver database, and 4) a focus on research and education. However, the depth, reach, and sustainability of these initiatives vary, presenting opportunities for further planning and investment.  Key areas for growth include 1) increasing caregiver awareness, 2) supporting employed caregivers, 3) improving program evaluation, 4) expanding public-private partnerships, and 5) workforce development. Research implications in California include exploring ways to further align existing caregiving infrastructure with national priorities to ensure that services are accessible, inclusive, and responsive to caregiver needs.

